# The alternative complement pathway is dysregulated in patients with chronic heart failure

**DOI:** 10.1038/srep42532

**Published:** 2017-02-14

**Authors:** Negar Shahini, Annika E. Michelsen, Per H. Nilsson, Karin Ekholt, Lars Gullestad, Kaspar Broch, Christen P. Dahl, Pål Aukrust, Thor Ueland, Tom Eirik Mollnes, Arne Yndestad, Mieke C. Louwe

**Affiliations:** 1 Research Institute of Internal Medicine, Oslo University Hospital, Rikshospitalet, Oslo, Norway; 2 Institute of Clinical Medicine, University of Oslo, Oslo, Norway; 3 Center for Heart Failure Research, University of Oslo, Oslo, Norway; 4 K.G. Jebsen Inflammation Research Centre, University of Oslo, Oslo, Norway; 5 Department of Immunology, Oslo University Hospital, Rikshospitalet, Oslo, Norway; 6 Linnaeus Centre for Biomaterials Chemistry, Linnaeus University, Kalmar, Sweden; 7 Department of Cardiology, Oslo University Hospital, Rikshospitalet, Oslo, Norway; 8 Section of Clinical Immunology and Infectious Diseases, Oslo University Hospital, Rikshospitalet, Oslo, Norway; 9 Research Laboratory, Nordland Hospital, Bodø, and K.G. Jebsen TREC, University of Tromsø, Tromsø, Norway; 10 Centre of Molecular Inflammation Research, Norwegian University of Science and Technology, Trondheim, Norway

## Abstract

The complement system, an important arm of the innate immune system, is activated in heart failure (HF). We hypothesized that HF patients are characterized by an imbalance of alternative amplification loop components; including properdin and complement factor D and the alternative pathway inhibitor factor H. These components and the activation product, terminal complement complex (TCC), were measured in plasma from 188 HF patients and 67 age- and sex- matched healthy controls by enzyme immunoassay. Our main findings were: (i) Compared to controls, patients with HF had significantly increased levels of factor D and TCC, and decreased levels of properdin, particularly patients with advanced clinical disorder (i.e., NYHA functional class IV), (ii) Levels of factor D and properdin in HF patients were correlated with measures of systemic inflammation (i.e., C-reactive protein), neurohormonal deterioration (i.e., Nt-proBNP), cardiac function, and deteriorated diastolic function, (iii) Low levels of factor H and properdin were associated with adverse outcome in univariate analysis and for factor H, this was also seen in an adjusted model. Our results indicate that dysregulation of circulating components of the alternative pathway explain the increased degree of complement activation and is related to disease severity in HF patients.

Chronic inflammation is involved in the pathogenesis of heart failure (HF), a disorder characterized by progressive loss of cardiac function with high morbidity and mortality^1^. Patients with HF are characterized by increased systemic and myocardial inflammation and experimental studies suggest that inflammatory mediators may be directly involved in maladaptive processes within the myocardium, promoting the development and progression of HF^2^.

The complement system is not only a host defence system against microbes, but also a surveillance system that contributes to maintain tissue homeostasis and tissue repair. However, uncontrolled complement activation can induce tissue damage, and inappropriate activation is involved in the pathogenesis of various conditions like autoimmune diseases, sepsis and transplant rejection^3^. Complement has also been demonstrated to play a role in myocardial ischemia-reperfusion damage and atherosclerosis formation^3^,^4^. The exact mechanisms of these detrimental effects are not fully elucidated, but likely involve tissue damage and activation of down-stream inflammatory processes^3^,^4^.

The complement system can be activated through three different pathways: the classical, the lectin and the alternative pathway (AP) which all lead to formation of the central C3- and C5-convertases, and finally forms the terminal C5b-9 complement complex (TCC). The AP is continuously undergoing a low-grade activation and functions as an amplifier of other routes of complement activation^3^. Key regulators of the AP are properdin and factor H (FH)^5^. Properdin stabilizes the alternative C3 convertase by binding to the labile C3bBb complex leading to C3bBbP, and has thereby an enhancing effect on the activation. In contrast, FH inhibits the formation of the AP convertase by binding to C3-fragments. Another important player is complement factor D (FD) which circulates in an active form and cleaves complement factor B (FB) to Ba and Bb^6^ thereby promoting AP activation (Fig. 1).

We and others have previously shown that the complement system is activated in patients with HF^7^, but data on regulation of the AP in HF is lacking. We hypothesized that an imbalance in components of the AP of complement activation, i.e., FH, FD and properdin, could be related to disease severity and HF progression.

## Results

### Circulating levels of alternative pathway complement components are altered and activation of complement is increased in HF patients

**Figure 2:**
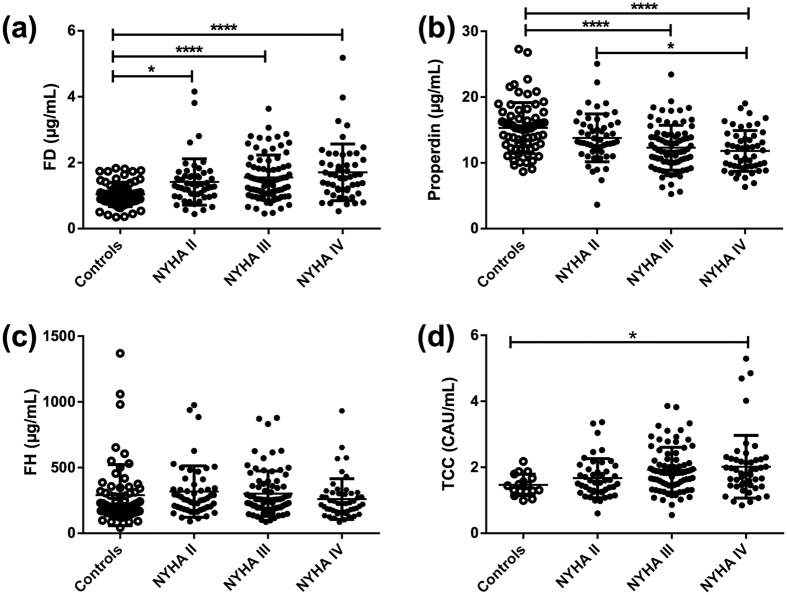
Plasma levels of alternative complement pathway components are altered and complement is activated in patients with heart failure. ELISA were used to measure plasma levels of complement factor D (FD) (**a**), properdin (**b**), factor H (FH) (**c**) and the terminal complement complex (TCC) (**d**) in 188 heart failure patients and 67 controls. NYHA, New York Heart Association functional class; TCC, terminal complement complex; Values are mean ± SD *p < 0.05, ****p < 0.0001.

To investigate if HF patients have altered levels of AP components, plasma of 188 patients with HF and 67 healthy controls were analyzed (Table 1). Levels of FD, the rate-limiting component of the AP, were elevated in HF patients as compared to controls, with particularly high levels in those with the more advanced clinical disease i.e., NYHA class III and IV (Fig. 2a). Conversely, levels of properdin, the stabilizer of the AP C3-convertase, were reduced in HF patients, and similar to FD, this alteration was particularly marked in patients with NYHA classes III and IV (Fig. 2b). Levels of FH, a negative regulator of the AP C3-convertase, were not different when comparing HF patients with healthy controls (Fig. 2c). Finally, levels of TCC were increased in HF patients with NYHA class IV as compared to controls confirming that the complement cascade had been activated to the very end in HF patients with the most advanced clinical disease (Fig. 2d).

### Circulating levels of FD, properdin and FH are associated with measures of cardiac function

**Figure 3:**
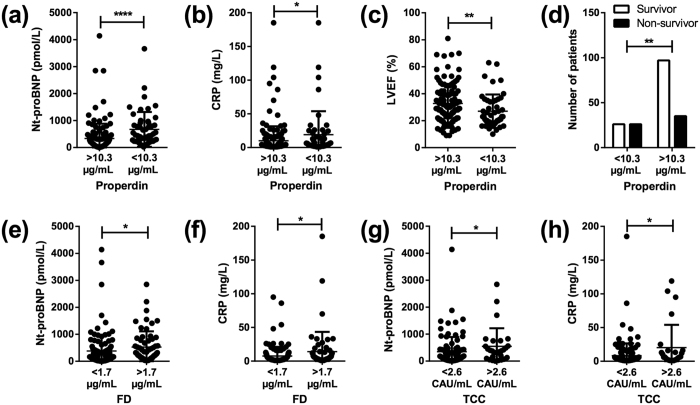
Heart failure patients with altered levels of alternative complement pathway components have more severe clinical manifestations. The 10–90 percentile ranges for properdin, factor D (FD) and TCC in healthy controls were calculated. Heart failure patients were dichotomized based on levels above or below 90 percentile in healthy controls for properdin, and below or above 10 percentile for FD and TCC, respectively. Levels of Nt-proBNP (**a**,**e**,**g**) and CRP (**b**,**f**,**h)** as well LVEF (**c**) and mortality (**d**) were compared in the two heart failure patient groups. TCC, terminal complement complex. Proportional difference in mortality was determined using Fisher’s exact test. Values are mean ± SD *p < 0.05, **p < 0.01, ****p < 0.0001.

To look in more detail into the clinical characteristics of HF patients with the lowest levels of properdin and the highest levels of FD and TCC, HF patients were dichotomized based on levels of TCC and FD above or below 90 percentile in healthy controls, and for properdin on levels below or above 10 percentile in healthy controls. These analyses showed that for HF patients with reduced properdin levels (i.e., below 10.3 μg/mL) elevated levels of Nt-proBNP, a marker of myocardial wall stress, and C-reactive protein (CRP), an inflammatory response marker, were observed, as well as a reduced left ventricular ejection fraction (LVEF) (Fig. 3a–c). In addition, reduced properdin levels were associated with markedly increased mortality (Fig. 3d). Furthermore, HF patients with elevated FD levels (i.e., above 1.7 μg/mL), and TCC levels (i.e., above 2.6 CAU/mL), had significantly higher Nt-proBNP and CRP levels (Fig. 3e–h), but had the same LVEF and clinical outcome as HF patients with normal FD levels (data not shown).

**Figure 4:**
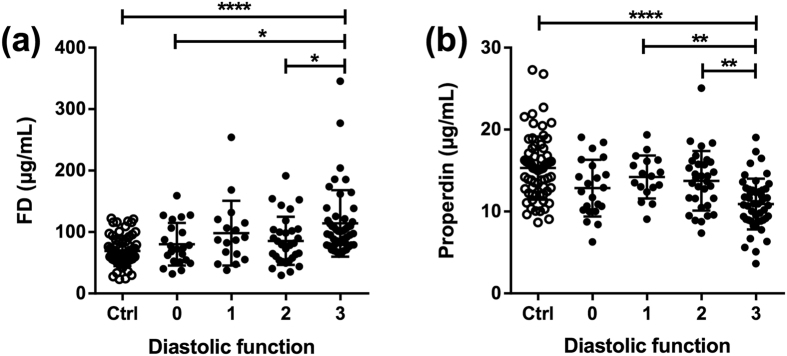
Diastolic function is associated with dysregulation of factor D and properdin. Plasma levels of complement factor D (FD) (**a**) and properdin (**b**) in patients with heart failure according to diastolic function as assessed by echocardiography. Diastolic dysfunction was defined as: 0, normal; 1, abnormal relaxation pattern; 2, pseudonormal filling dynamics; 3, restrictive filing dynamics. Values are mean ± SD *p < 0.05, **p < 0.01, ****p < 0.0001.

**Figure 5:**
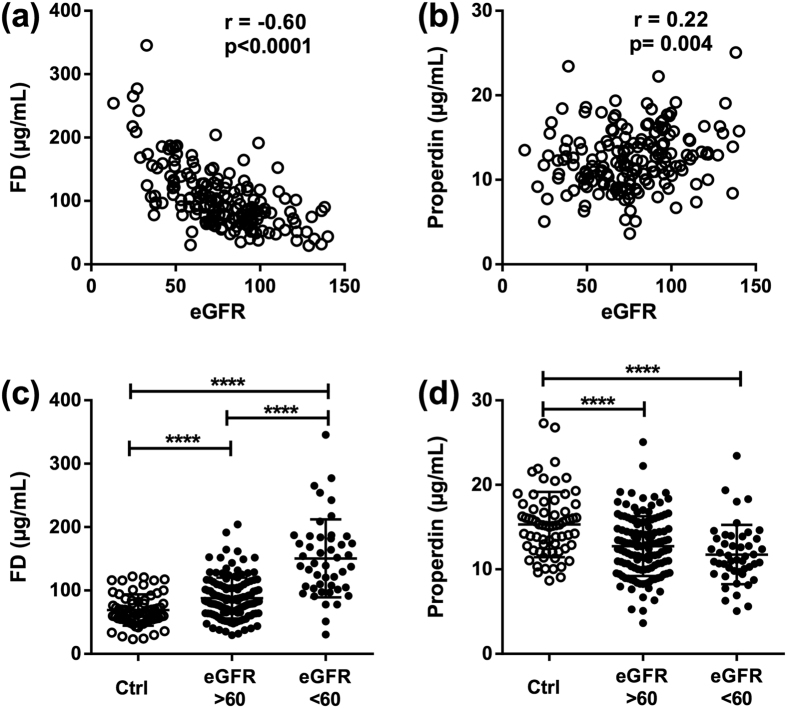
Factor D and properdin are associated with impaired kidney function. Correlation between estimated glomerular filtration rate (eGRF) and plasma levels of factor D (FD) (**a**) and properdin (**b**). Levels of FD (**c**) and properdin (**d**) in healthy controls and in HF patients with normal (i.e. eGFR > 60) or deteriorated (i.e. eGFR < 60) kidney function. Values are mean ± SD ****p < 0.0001.

When further analyzing the relationship between complement components and clinical and biochemical markers of cardiac function, several significant findings were revealed (Table 2). First, levels of all measured AP regulators were significantly correlated with levels of Nt-proBNP with a negative correlation for properdin and FH, and a positive correlation for FD. Second, low properdin levels were associated with impaired systolic function (i.e., LVEF and cardiac output (CO)). Third, when grading diastolic function (Fig. 4 and Table 2), we found particularly high levels of FD (Fig. 4a) and low levels of properdin (Fig. 4b) in HF patients with the most restrictive filling dynamics. Fourth, FD was positively correlated with CRP, whereas properdin was negatively correlated with CRP. Fifth, in particular FD but also properdin were significantly correlated with eGFR (Fig. 5a,b and Table 2), indicating that impairment of kidney function has an impact on the plasma levels of these factors. Importantly, however, when we compared levels of FD (Fig. 5c) and properdin (Fig. 5d) in patients with preserved kidney function (i.e., eGFR > 60 mL∙min^−1^ ∙1.73 m^−2^) with control subjects, we still found significantly changed levels in HF, indicating that altered levels of these factors do not merely reflect impaired kidney function. While there was not any correlation between FH and properdin and liver function, the measure of FD was inversely correlated with measures of liver damage as assessed by plasma levels of alanine aminotransferase (Table 2). Lastly, FD was positively correlated with FH and negatively correlated with properdin whereas no significant correlations were found between the other complement factors (Table 2).

### Baseline properdin and FH levels predict long term mortality in HF patients

**Figure 6:**
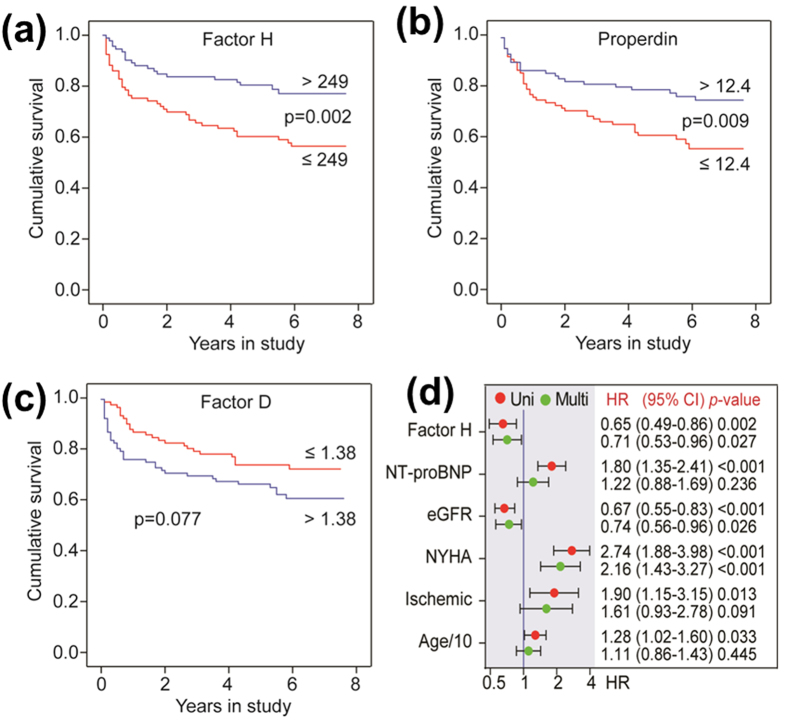
Factor H and properdin levels are associated with all-cause mortality in heart failure. Kaplan-Meier survival curves in relation to dichotomized levels (blue line, high and red line, low) of factor H (**a**), properdin (**b**) and factor D (**c**) using all-cause mortality/heart transplantation as end point. (**d**) Hazard ratios based on dichotomized factor H levels, estimated by Cox proportional analysis. Uni is unadjusted and Multi is adjusted for N-terminal pro-B-type natriuretic peptide (Nt-proBNP), estimated glomerular filtration rate (eGFR), New York Heart Association functional class (NYHA), ischemia and age per 10 years. Results of univariate and multivariate Cox proportional analyses of the adjustment variables are shown for reference.

During a mean follow up of 4.6 ± 2.3 years, 60 patients experienced adverse outcome; 48 patients died and 12 underwent heart transplantation. Kaplan-Meier survival analyses demonstrated that low levels (i.e., below median) of FH (Fig. 6a) and properdin (Fig. 6b), but not FD (Fig. 6c) were associated with adverse outcome. The hazard ratios (HR) were 0.65 and 0.72 for FH and properdin, respectively, in univariate cox regression (Fig. 6d, properdin not shown). Multivariable adjustment for covariates (Nt-proBNP, eGFR, NYHA, ischemic aetiology and age) attenuated these associations but remained significant for FH (Fig. 6d, HR = 0.71, p = 0.027).

### Elevated levels of FD enhance AP activation and depletion of properdin is consistent with surface binding

**Figure 7:**
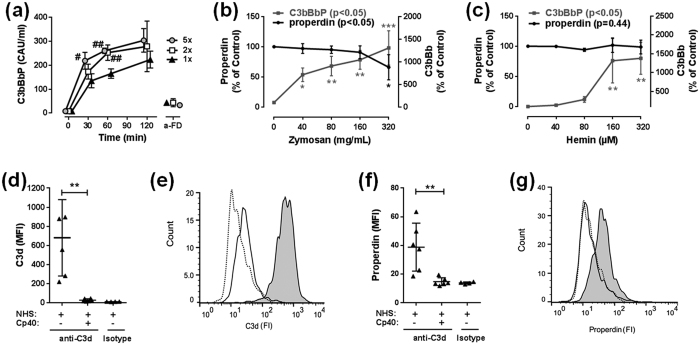
*Ex vivo* studies on alternative pathway complement activation. Normal human serum (NHS) was incubated *ex vivo* with different doses of FD (**a**), zymosan (**b**) and hemin (**c**) particles. Levels of C3bBbP and/or properdin were determined in three independent experiments by enzyme immunoassays. (**d**–**g**), NHS was incubated on hypoxic endothelial cells with or without the C3 inhibitor compstatin (Cp40) and surface deposition of C3d (**d**) and properdin (**f**) was determined by flow cytometry. (**e**,**g**) Show flow cytometry histogram, representative from one of six (NHS +/− Cp40) or four (isotype control) experiments. NHS is represented in filled grey, NHS + Cp40 in filled white and antibody isotype control with dotted lines, for C3d (**e**) and properdin (**g**) respectively. Values are mean ± SD ^#^p < 0.05, ^##^p < 0.01, vs. 1×; *p < 0.05, **p < 0.01, ***p < 0.001 vs. properdin.

FD levels were increased in HF patients and related to a decreased survival. Since FD is generally accepted to be a rate-limiting factor of the AP^8^, we investigated to what extent elevated FD contributes to increased AP activation. We studied basal, 2 times and 5 times elevated FD levels *ex vivo* in normal human serum (NHS) for up to 120 minutes (Fig. 7a). The C3bBbP complex, as a marker for AP activation, was spontaneously formed in NHS from 30 minutes and onwards. By increasing FD levels up to 5 times with purified protein, the levels of C3bBbP were, after 30 and 60 minutes incubation, significantly elevated. Addition of anti-FD completely reversed this effect, demonstrating specificity of the reaction.

In HF patients we observed that properdin was markedly decreased, and low levels were significantly associated with clinical, neurohormonal and echocardiographic measures of disease severity, as well as poor prognosis. To address the mechanism for the decreased properdin levels in HF patients, we examined properdin levels in two different *ex vivo* models, triggering complement activation in NHS, on solid-phase by zymosan particles^9^ (Fig. 7b) or in fluid-phase by hemin^10^ (Fig. 7c). Both activators contribute to fluid-phase complement activation, but zymosan, in contrast to hemin, also represent a surface which may bind complement components and activation fragments. Zymosan as well as hemin increased levels of fluid-phase C3bBbP (Fig. 7b and c, respectively). However, whereas properdin levels decreased when incubated with zymosan (Fig. 7b) it remained unchanged when incubated with hemin (Fig. 7c), suggesting that the observed reduction of properdin is associated with binding of properdin to the activating surface, here zymosan. Next, NHS was incubated on hypoxia stressed endothelial cells (ECs), as a model of blood-endothelial cell interaction in underperfused tissue (Fig. 7d–g). As expected, C3d deposition, indicating complement activation, was detected on the hypoxic EC (Fig. 7d) and in parallel, properdin was also found to bind to these cells (Fig. 7f). Addition of the C3-inhibitor compstatin Cp40 fully inhibited the deposition of both C3d and properdin (Fig. 7d and f, respectively). Thus, blocking complement activation inhibits binding of properdin to the EC surface, suggesting a shift in properdin from the fluid phase to the tissue phase during complement activation.

## Discussion

The complement system is activated during HF and uncontrolled activation of this system can induce extensive inflammation and tissue damage. However, it is unknown to which extent the AP contributes to this. In this study we investigated specifically the central regulators of the AP, i.e., properdin, FH and FD, in plasma from patients with HF. Our main findings were that patients with HF had elevated levels of FD, but lower levels of properdin, together implying a hyper-activity in AP, and these changes were related to HF severity. Furthermore, low levels of properdin and FH were associated with impaired long-term transplant-free survival. At last, our *ex vivo* experiments suggest that the reduced properdin levels in HF patients reflect enhanced complement activation due to a shift of this molecule from the fluid-phase to tissue.

Others and we have previously demonstrated that complement activation occurs in chronic HF and this is associated with adverse clinical events^7^,^11^. Moreover, we recently showed that complement activation can occur through the lectin pathway (i.e., ficolin-3) in HF^12^. The complement convertases C3 and C5 are studied most in relation to HF, as these are the common mediators of complement activation through all three activation pathways^13^. Recently C3c, a stable C3-conversion product, has been proposed as a suitable biomarker for patients with HF^14^. In the present study we show that HF patients are also characterized by an increased activation potential of the AP, as demonstrated by increased plasma levels of FD that promotes AP activation and is significantly associated with clinical and neurohormonal disease severity. The AP convertase inhibitor FH was not elevated in HF patients, however, low levels (i.e., ≤249 μg/mL) were associated with impaired transplant-free survival during follow-up, even in the adjusted analyses, further suggesting a link between AP activation and HF severity.

FD is thought to be one of the rate-limiting proteins in the AP and our *ex vivo* study corroborates this. We showed spontaneous formation of the C3bBbP complex in serum, and increased levels of FD result in even higher C3bBbP levels. This finding underlines that the AP convertase can be generated more rapidly by spontaneous C3 hydrolysis if active FD is available.

Properdin is a regulator of the AP which enhances the activation of the system, and somewhat surprisingly we found that properdin levels were reduced in HF patients. Moreover, low levels of properdin (i.e. ≤12.4 μg/mL) were associated with parameters of clinical and neurohormonal disease severity, and at least in the unadjusted analyses, low properdin levels were associated with low transplant-free survival. This may seem to be in contradiction with a recent study of complement system regulators in patients in risk of developing cardiovascular disease showing increased levels of properdin that also were associated with cardiovascular events^15^. However, our interpretation of this discrepancy is that it illustrates the role of complement in different stages of cardiovascular disease, from early asymptomatic disorders to end-stage HF, as is the case for many of the patients in our cohort. More in line with the results of our study is a recent report of patients with sepsis, a severe systemic disorder, who had markedly decreased properdin levels associated with inflammation^16^. Our *ex vivo* studies further support that low systemic properdin levels could reflect markedly enhanced complement activation. By activating serum *ex vivo* with monomeric hemin or zymosan particles, we showed that in serum stimulated with zymosan, exhibiting a solid phase for activation, properdin levels were reduced in the fluid phase together with increased levels of C3bBb as a marker of AP activation. Moreover, incubation of serum on hypoxic endothelial cells showed increased deposition properdin on the cell surface. Altogether this suggests that during enhanced complement activation, that also includes AP activation, properdin levels in plasma are decreased due to binding to the surface (e.g. EC), thus reflecting a shift from the fluid to the tissue for this mediator of enhanced AP activation.

Interestingly, dysregulation of properdin and FD was associated with diastolic dysfunction. The reason for this association is not clear, but as diastolic dysfunction is related to systemic inflammation, often associated with inflammatory co-morbidities, the association of AP activation to diastolic dysfunction could reflect inflammation as a common feature^17^. Moreover, enhanced myocardial fibrosis is another feature of diastolic dysfunction and enhanced complement activation including AP activation has been associated with renal fibrosis in lupus nephritis^18^ and inflammation and fibrosis in a model of experimental colitis^19^. Also, although not investigated for AP activation, activation of the lectin pathway have been suggested to play a pathophysiological role in hyperglycaemia-induced cardiac fibrosis, systolic and diastolic dysfunction in experimental model of acute hyperglycaemia^20^. It is tempting to hypothesize that the combined role of the AP in inflammation and fibrogenesis may be of particular importance for the development of diastolic dysfunction. This has to be examined in forthcoming larger clinical studies analyzing AP parameters in patients with HF with preserved LVEF [HFpEF] and experimental studies, particularly focused on mechanisms that can explain the association between the dysregulation of FD and properdin *and* diastolic dysfunction.

There are some limitations of the current study. Our results were derived from a selected patient population with moderate to severe chronic HF. The patients were all referred to a tertiary hospital for evaluation. Therefore, extrapolation of our results to a broader HF population should be done with caution. An additional limitation is the lack of specific cause of death which allows analyzing all-cause mortality only. The study population was relatively small, so although interesting correlations and relationships to mortality were found, they should be interpreted carefully.

In conclusion, our results show that the AP is activated in HF as levels of promoters and inhibitors of the pathway are altered in HF patients. Dysregulation of the AP is related to disease severity and the association to diastolic dysfunction may be of particular interest, potentially reflecting the combined involvement of AP activation in inflammation and fibrogenesis.

## Methods

### Study population

One hundred eighty-eight patients with chronic stable HF in New York Heart Association (NYHA) functional class II–IV were consecutively included in the study (Table 1). The aetiology of HF was determined by patient history, echocardiography, and coronary angiography and classified as coronary artery disease (CAD, n = 78), dilated cardiomyopathy (DCM, n = 99), or other (n = 9), information from two patients was absent. More detailed information of these patients is given by Norum *et al*.^21^. For comparison, blood samples were collected from 67 sex- and age-matched healthy control subjects (22 females and 45 males). A combination endpoint for survival analysis consisting of all-cause mortality and heart transplantation was used.

The clinical parts of this study were approved by the local ethical committee (Regional ethics committee of Helse Sør-Øst; Permit number S-05172) and conducted according to the ethical guidelines outlined in the Declaration of Helsinki for use of human tissue and subjects. Informed written consent was obtained from each participant.

### Echocardiography

Echocardiographic imaging of the heart was obtained from apical and parasternal views with the use of a GE Vivid 7 ultrasonic digital scanner (GE Vingmed, Horten, Norway). Conventional 2-dimensional images, M-mode, colour Doppler, and pulsed-wave Doppler recordings of blood flow through cardiac ostia were obtained^22^. Echocardiographic data were analyzed offline with GE Echopac BT 10 software. Left ventricular ejection fraction (LVEF) was calculated from apical imaging views with the use of the modified biplane Simpson method^22^. Based on the ratio of early to late transmitral diastolic filling velocity (E/A) we applied a modified version of the scheme proposed by Appleton *et al*.^23^ for grading diastolic function (0, normal; 1, abnormal relaxation pattern; 2, pseudo normal filling dynamics; 3, restrictive filling dynamics) in patients with sinus rhythm, as recommended by the American Society of Echocardiography^22^. Our study population had a wide age span, and we therefore used age-specific cut off values for E/A ratios based on a Norwegian survey for normal echocardiographic values^24^.

### Right-Sided Catheterization

A Swan-Ganz pulmonary artery thermo dilution catheter (Baxter Health Care Corp, Santa Ana, California) was used to perform right-sided heart catheterization. Pulmonary artery pressures were recorded. Cardiac output (CO) was measured by means of the thermo dilution technique.

### Biochemistry and blood sampling

Peripheral venous blood was drawn into pyrogen-free EDTA tubes, immediately immersed in melting ice, and centrifuged within 30 minutes at 2000 *g* for 20 minutes, and platelet-poor plasma was stored in multiple aliquots at −80 °C. The samples were thawed three times or less before analyses. C-reactive protein (CRP) and N-terminal pro–B-type natriuretic peptide (Nt-proBNP) were analyzed with the use of the Modular platform (Roche Diagnostics, Basel, Switzerland).

### Measurements of circulating complement factors in HF patients

Enzyme-linked immunosorbent assays (ELISA) was used to measure circulating levels of FD (plasma diluted 1:15000; Catalog #DY1824, R&D Systems, Minneapolis, MN), properdin (plasma diluted 1:5000; Catalog #HK334-01, Hycult Biotech, Uden, the Netherlands) and FH (plasma diluted 1:5000; Catalog #DY4779, R&D Systems, Minneapolis, MN). TCC (plasma diluted 1:5) was measured by an in-house ELISA as previously described^25^. The results are given in complement arbitrary units (CAU) per mL, related to a standard that was made by zymosan activated serum containing 1000 CAU/mL.

### *Ex vivo* model for FD induced AP complement activation

Normal human serum (NHS), with or without (1x) purified FD (2×, i.e. 1.5 μg/mL and 5×, i.e. 6 μg/mL; Complement Technology Inc, Tyler, TX), was incubated at 37 °C for 30, 60 and 120 minutes. A mouse anti-human FD (clone 166-32)^26^ was included in separate tubes and incubated for 120 min in concentrations corresponding to the levels of FD (i.e. 40, 80, 200 μg/mL). The C3bBbP complex was measured as an indicator of AP complement activation by an in-house ELISA^25^.

### *Ex vivo* models for fluid-phase and surface induced complement activation

NHS was incubated at 37 °C for 30 minutes with different concentrations of hemin (0–320 μM; Sigma-Aldrich, St. Louis, MO) or zymosan (0–500 μg/mL; from *Saccharomyces cerevisiae*, Sigma-Aldrich, St. Louis, MO) as a fluid- and solid-phase activator of the complement system, respectively. Following incubation, EDTA was added (10 mM final concentration) and the samples were centrifuged at 3000 *g* for 20 minutes at 4 °C. Serum properdin levels were measured with the same kit as it used for HF patients. The C3bBbP complex was measured as an indicator of the complement AP activation by an in-house ELISA^25^.

### Complement activation and binding of properdin to hypoxic endothelial cells

Confluent human umbilical vein endothelial cells (HUVECs) in passages two through five, isolated and cultured as previously described^27^, were grown in complete endothelial cell culture medium (Cell Applications, San Diego, CA) and exposed to low oxygen (1%) for 16 hours using the Ruskinn *in vivo* 400 hypoxia incubator (Ruskinn Technologies, Bridgend, UK). The cells were washed with sterile PBS pH 7.4 and reoxygenated (21% O_2_) for four hours with NHS with or without the C3 inhibitor compstatin Cp40 (20 μM; kindly provided by prof. John Lambris, University of Pennsylvania). Then, the cells were washed twice in PBS and fixed in 0.5% paraformaldehyde for 2.5 minutes. Deposition of C3d was detected using mouse anti-C3d clone 7C10 (Abcam, Cambridge, UK), binding of properdin with mouse anti-human properdin IgG1κ (A233) (Quidel Corporation, San Diego, CA). Clone IS7 (IgG1κ, anti-human CD22; Diatec Monoclonals AS,Oslo, Norway) was used as isotype control and FITC-labelled goat anti-mouse IgG1 (Southern Biotech, Birmingham, AL) was used as secondary antibody to all three detection antibodies. Cells were trypsinated and analyzed on a FACSCalibur flow cytometer (BD Biosciences, San Jose, CA). Data was analyzed in FlowJo X (Tree Star, Ashland, OR).

### Statistical Analyses

Differences between groups were analyzed with the use of Mann-Whitney *U* tests or Student *t*-tests, as appropriate. When comparing ≥3 groups, one-way analysis of variance (ANOVA) or the Kruskal-Wallis test was used for continuous data, depending on distribution (Kolmogorov-Smirnov test). Associations between variables were assessed by means of Spearman correlation coefficient. The Shapiro-Wilk test was performed to assess data distribution before inclusion in regression models. Variables that were not normally distributed were log transformed before statistical analyses. Kaplan-Meier analysis with log rank test was used to analyze all-cause/anticipated mortality stratified by dichotomized levels of FH and properdin. Cox proportional hazard analysis was performed to estimate hazard ratios (HRs) adjusting for age, sex, NYHA functional class, and etiology. Subsequently, adjustments were made for CRP and Nt-proBNP. P-values are 2-sided and considered to be significant when <0.05.

## Additional Information

**How to cite this article:** Shahini, N. *et al*. The alternative complement pathway is dysregulated in patients with chronic heart failure. *Sci. Rep.* **7**, 42532; doi: 10.1038/srep42532 (2017).

**Publisher's note:** Springer Nature remains neutral with regard to jurisdictional claims in published maps and institutional affiliations.

## Figures and Tables

**Figure 1:**
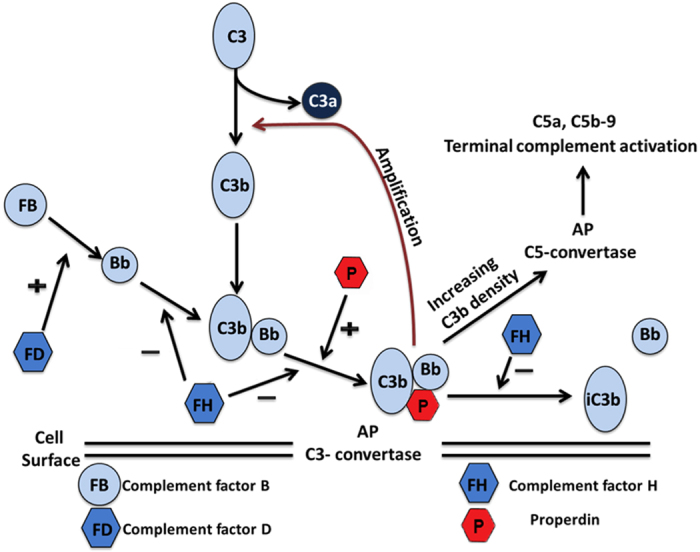
Activation of the alternative complement pathway. The figure summarizes the activation of the alternative pathway with the main components C3 and factor B (FB), the activator of FB, factor D (FD), and the regulators of AP activation: the inhibitor factor H (FH) and the enhancer properdin.

**Table 1:** Characteristics of the study population.

	Controls (n = 67)	Total population (n = 188)	Survival (n = 127)	Non-survival (n = 61)
*Clinical characteristics*				
Male sex, n (%)	45 (67)	148 (79)	101 (80)	47 (77)
Age, y	60 ± 7.2	55.7 ± 12.7	54.2 ± 12.8	58.8 ± 12.2
BMI, kg/m^2^	24.7 ± 2.4	26.0 ± 5.8	26.6 ± 5.9	25.9 ± 5.4
Etiology (CAD/DCM/other), n		78/99/9	44/75/6	34/24/3
Duration, y		5.1 ± 6.2	4.1 ± 5.1	7.0 ± 7.7^##^
NYHA: II/III/IV, n		53/81/51	49/52/23	4/29/28
Type 2 DM, n (%)		25 (13)	16 (13)	9 (15)
Hypertension, n (%)		29 (15)	18 (14)	11 (18)
Previous MI, n (%)		64 (34)	38 (30)	26 (43)
COPD, n (%)		20 (11)	15 (12)	5 (8)
*Echocardiography*				
LVEF, %		31.0 ± 13.8	31.8 ± 14.2	29.5 ± 13.1
CO, L/min		4.0 ± 1.3	4.1 ± 1.3	3.8 ± 1.3
CI, L/min/m^2^		2.0 ± 0.6	2.0 ± 0.6	1.9 ± 0.5
*Biochemistry*				
WBC, 10^9^/L	5.5 ± 1.3	7.5 ± 2.5***	7.4 ± 2.5***	7.6 ± 2.6***
eGFR, mL min^−1^ 1.73	92.7 ± 12.2	77.5 ± 28.0***	82.0 ± 25.6*	68.4 ± 29.7***^##^
ALT	23 [18, 28]	25 [19, 39]	27 [19, 43]	23 [18, 32]
Cholesterol, mmol/L	5.8 ± 0.8	4.1 ± 1.2***	4.4 ± 1.2***	3.7 ± 1.0***^##^
NT-proBNP, pmol/L	4.9 [3.1, 8.7]	234 [81, 476]	182 [49, 413]	312 [205, 750]
CRP, mg/L	1.2 [0.8, 2.4]	3.3 [1.6, 7.9]	3.0 [1.3, 6]	4.8 [2, 11.9]
*Medication, n (%*)				
ACE inhibitors		127 (67)	88 (70)	39 (63)
ARBs		40 (21)	22 (17)	18 (29)
β-Blockers		151 (80)	102 (81)	49 (79)
Diuretics		134 (71)	82 (65)	52 (84)
Warfarin		81 (43)	45 (36)	36 (58)
ASA		79 (42)	58 (46)	21 (34)
Statins		82 (43)	48 (38)	34 (55)

BMI, body mass index; CAD, coronary artery disease; DCM, dilated cardiomyopathy; NYHA, New York Heart Association functional class; DM, diabetes mellitus; MI, myocardial infarction; COPD, chronic obstructive pulmonary disease; LVEF, left ventricular ejection fraction; CO, cardiac output; CI, cardiac index; WBC, white blood cell count; eGFR, estimated glomerular filtration rate; NT-proBNP, N-terminal pro–B-type natriuretic peptide; CRP, C-reactive protein; ACE, angiotensin-converting enzyme; ARB, angiotensin receptor blocker; ASA, acetylsalicylic acid. Values are presented as number (%), mean ± SD, or median [interquartile range]. ***p < 0.001 vs controls, ^##^p < 0.01 vs survivors.

**Table 2:** Associations of FH, FD and properdin with heart function and biochemical parameters.

	FD	FH	Properdin
Nt-proBNP	0.27 **P < 0.001**	−0.19 **P = 0.008**	−0.44 **P < 0.001**
LVEF	−0.05 P = 0.49	0.10 P = 0.18	0.22 **P = 0.003**
LVEDV	−0.07 P = 0.35	−0.03 P = 0.68	−0.03 P = 0.64
LVESV	−0.04 P = 0.55	−0.04 P = 0.56	−0.09 P = 0.23
CO	0.04 P = 0.54	0.09 P = 0.23	0.18 **P = 0.01**
PCWP	0.26 **P = 0.007**	−0.16 P = 0.09	−0.23 **P = 0.01**
Diastolic function	0.29 **P = 0.002**	0.013 P = 0.89	−0.31 **P < 0.001**
E/A ratio	0.11 P = 0.24	0.02 P = 0.81	−0.34 **P < 0.001**
CRP	0.19 **P = 0.01**	−0.07 P = 0.29	−0.26 **P < 0.001**
eGFR	−0.59 **P < 0.001**	0.05 P = 0.51	0.27 **P < 0.001**
ALT	−0.18 **P = 0.026**	−0.06 P = 0.43	0.04 P = 0.66
FD	—	0.30 P < 0.0001	0.24 P = 0.003

FD, complement factor D; FH, complement factor H, Nt-ProBNP, N-Terminal pro-B-type natriuretic peptide; LVEF, left ventricular ejection fraction; LVEDV, left ventricular end-diastolic volume; LVESV, LV left ventricular end-systolic volume; CO; cardiac output, PCWP, pulmonary capillary wedge pressure; CRP, C-reactive protein; eGFR, estimated glomerular filtration rate; ALT alanine aminotransferase;. P-values < 0.05 are in bold.
